# Management capacity for stable coronary heart disease in Shanghai community medical institutions: a cross-sectional study

**DOI:** 10.1186/s12913-025-13486-y

**Published:** 2025-10-07

**Authors:** Sen Yang, Liuhua He, Jiaojiao Wang, Le Ma, Wanyu Li, Yang Wang, Bosheng Mei, Hanzhi Zhang, Ying Pan, Dehua Yu, Hua Jin

**Affiliations:** 1 Daqiao Community Healthcare Centre, Yangpu District, Shanghai, 200090 PR China; 2 Department of General Practice, Yangpu Hospital, School of Medicine, Tongji University, Shanghai, 200090 China; 3 School of Medicine, Tongji University, Shanghai, 200331 China; 4 Huayang Street Community Health Service Centre, Changning District, Shanghai, 200042 China; 5 Shanghai General Practice Clinical Quality Control Centre, Shanghai, 200090 China; 6 Xiayang Street Community Health Service Centre, Qingpu District, Shanghai, 201703 China; 7 Department of Emergency Surgery, Union Hospital, Tongji Medical College, Huazhong University of Science and Technology, Wuhan, 430022 China; 8 Shanghai General Practice and Community Health Development Research Centre, Shanghai, 200090 China

**Keywords:** Coronary heart disease, Diagnostic quality, Therapeutic quality, Management capacity, Primary healthcare, Quality assessment, Health services evaluation

## Abstract

**Background:**

Coronary heart disease (CHD) remains one of the leading causes of death worldwide. However, systematic evaluations of CHD management quality at the community level remain limited, thereby constraining improvements in primary medical capacity. This study aims to evaluate community-based CHD management using Donabedian’s model to optimise resource allocation, standardise clinical pathways, and improve chronic disease management.

**Methods:**

Guided by Donabedian’s model, this study assessed the quality of CHD diagnosis and management within Shanghai’s primary healthcare system across three dimensions—structure, process, and outcome—from the dual perspectives of community healthcare institutions and general practitioners (GPs). A cross-sectional survey was conducted between April and May 2024, involving 247 primary healthcare institutions selected through census sampling. Within each administrative district, 50% of institutions were randomly selected using cluster sampling. Subsequently, stratified sampling based on professional titles was employed to survey 50% of GPs within these institutions. In total, 247 institutional questionnaires and 2,093 GPs’ responses were deemed valid and included in the final analysis.

**Results:**

Structural analysis indicated adequate CHD-specialised clinics (74.8% with integrated care teams) and essential equipment availability (> 97%), but significant gaps in rehabilitation resources (personnel: 14.6%; equipment: 8.5%). Process evaluation showed high referral rates (91.5%) yet poor patient self-management (18.6%) and limited health record completeness (27.1%). Only 26.7% of institutions adopted CHD-specific information systems. GPs demonstrated strong acute care capacity but had knowledge gaps in advanced concepts (53.6%) and test interpretation (45.4%). Key barriers included equipment shortages (75.5%) and protocol adherence issues (73.1%), with prioritised solutions emphasising medical consortium collaboration (89.4%) and multidisciplinary team development (88.3%).

**Conclusion:**

CHD management in Shanghai’s community settings is marked by adequate provision of essential resources but notable deficiencies in rehabilitation services and digital infrastructure. Strengthening rehabilitation services, enhancing information system development, and providing targeted training to improve diagnostic and management capacities are recommended. These findings may provide valuable insights for informing similar efforts in other regions.

**Supplementary Information:**

The online version contains supplementary material available at 10.1186/s12913-025-13486-y.

## Introduction

Coronary heart disease (CHD) is one of the leading causes of death globally [1–3]. According to the latest epidemiological survey, the current number of CHD patients in China has reached 11.39 million, with a persistent upward trend [4]. The CHD mortality rate among urban residents is 135.08 per 100,000, while it is even higher in rural areas at 148.19 per 100,000 [5]. This underscores the urgency and necessity of strengthening CHD prevention and control. With the advancement of the “Healthy China 2030” strategy, China has explicitly proposed a healthcare policy emphasising “primary care as the focus and prevention as the priority”, gradually shifting the emphasis of disease management from hospitals to communities [5]. As the frontline for chronic disease management and prevention, primary health care (PHC) institutions play a critical role in community-based CHD management [6, 7]. Studies have shown that community participation in CHD management significantly improves patients’ quality of life, reduces hospitalisation rates, and enhances overall management effectiveness [8].

Globally, diverse models of community-based CHD management have been established, with accumulated rich experience. For instance, the 2023 Chronic Coronary Disease Management Guidelines by the American Heart Association (AHA) emphasises a patient-centred, lifelong disease management approach, utilising risk stratification for individualised interventions and integrating electronic health record systems to enable cross-institutional data sharing [9]. The Atherosclerosis Risk in Communities (ARIC) study, initiated in 1987 in the United States, conducts community-based surveillance to understand trends in CHD incidence, mortality, and subclinical atherosclerosis, providing critical evidence for community management and prevention [7]. In Spain, mobile health applications in community CHD management have significantly improved patients’ adherence to Mediterranean diets, healthy food intake frequency, physical activity levels, and smoking cessation rates, effectively controlling cardiovascular risk factors and enhancing quality of life [10]. Germany’s “Heart Care Navigator” program, led by specialised nurses for post-discharge follow-ups, has demonstrated that care management interventions supporting patients’ transition from hospitalisation to outpatient care reduce readmission rates, shorten readmission durations, and improve adherence to post-discharge recommendations [11]. In Australia, studies investigating disparities in community management among CHD patients of different socioeconomic statuses have informed strategies for optimising resource allocation and improving management efficacy [12]. Additionally, community-participatory research methods have explored the role of communities in cardiovascular disease management, revealing that community engagement effectively enhances patient outcomes and reduces health inequities [8].

Despite the pivotal role of PHC institutions in CHD prevention and the significant progress in management practices globally [13], systematic evaluations of CHD diagnosis, treatment quality, and management capabilities at the community level remain scarce [14]. In China, particularly, the evaluation system for CHD care quality and management capacity in PHC institutions is underdeveloped, leaving a notable research gap. Since its establishment in 2018, the Shanghai Clinical Quality Control Centre for General Practice, where the research team is based, has built a two-tiered quality control network linking municipal and district levels and developed a standardised supervision system for clinical quality control in general practice [15]. The centre has continuously implemented comprehensive training and biannual quality supervision, adopting a cross-regional inspection model. Results are systematically analysed, summarised, and fed back to institutions, effectively assisting PHC facilities in identifying issues and implementing targeted improvements, thereby significantly enhancing the quality of general practice [16]. In the field of quality control research, the centre has conducted citywide studies on diseases such as Helicobacter pylori infection, Chronic Obstructive Pulmonary Disease (COPD), asthma, osteoporosis, and chronic kidney disease (CKD), accumulating extensive experience and producing multiple research outcomes [17, 18]. However, empirical research on the current status of CHD diagnosis and treatment quality and management capacity in PHC institutions remains limited, highlighting an urgent need for further in-depth investigation.

On this basis, this study selects Shanghai—a city with advanced economic development and a well-established community healthcare service system in China—as the research region. By systematically evaluating the quality of CHD diagnosis, treatment, and management capabilities in Shanghai’s communities, this research aims to provide empirical evidence for optimising healthcare resource allocation and formulating targeted intervention strategies. Furthermore, the findings may offer transferable insights for other regions, with significant practical implications for advancing the early-stage prevention and control of CHD.

## Materials and methods

### Study subjects

This study targeted 247 PHC institutions in Shanghai, systematically evaluating the current status of community-based CHD management from two dimensions: institutional management and general practitioners’ (GPs’) practices. A census method was applied to community health centres, covering all 16 administrative districts in Shanghai, with 247 PHC institutions included as a full-sample survey (details in Supplementary file 1). For community GPs, a multi-stage sampling method was adopted: Stage 1 (Cluster Random Sampling): 50% of PHC institutions were randomly selected from the 16 administrative districts. Stage 2 (Stratified Random Sampling): Licensed physicians within the sampled institutions were stratified into four levels based on professional titles—resident physicians, attending physicians, associate chief physicians, and chief physicians—with 50% of GPs randomly selected from each stratum. In China, GPs are classified into three primary professional title levels based on years of service, qualifications, and professional assessment: junior (e.g., Resident Physician), intermediate (e.g., Attending Physician), and senior (e.g., Associate Chief Physician or Chief Physician). In this study, we used these standard categories to describe the professional composition of participants.

### Inclusion Criteria

① PHC institutions in Shanghai are willing to participate.

② Community GPs currently employed and on duty.

③ Participants voluntarily signed informed consent after fully understanding the study’s purpose and content, committing to provide necessary cooperation.

This study was approved by the Ethics Committee of Yangpu Hospital Affiliated to Tongji University (Approval No.: LL-2025-LW-001).

### Methods

#### Survey tools

Based on the two levels of investigation targets (institutions and physicians), the survey included institution-specific and physician-specific questionnaires. Both questionnaires were developed in accordance with the Primary Care Guidelines for Stable Coronary Heart Disease (2020) [19] and the Primary Care Guidelines for Cardiac Rehabilitation in CHD (2020) [20], and were structured using the Donabedian quality assessment framework (Structure-Process-Outcome, SPO model) [21]. This study employs Donabedian’s Structure–Process–Outcome (SPO) model, which provides substantial practical value through its three-dimensional framework for systematically evaluating all aspects of PHC quality [22]. By examining the interrelationships among resource allocation (structure), diagnostic and treatment protocols (process), and core knowledge of CHD (as a proxy outcome indicator), the model offers a comprehensive analytical approach [23]. Given the limited accessibility of PHC outcome data, this study indirectly assesses diagnostic and treatment quality through the CHD knowledge levels of GPs. In contrast to the United Kingdom’s Quality and Outcomes Framework (QOF) [24], which emphasises performance incentives and outcome metrics, the SPO model is better aligned with the quality improvement needs of Shanghai’s PHC system, enabling a more nuanced analysis of current bottlenecks and potential development pathways in CHD management.

The present study encompasses the following dimensions: (1) Structure: Focused on institutions, assessing staffing for CHD speciality clinics, availability of diagnostic/therapeutic equipment, and medication supply. (2) Process: Targeted institutions, covering implementation programs, quality control, and IT infrastructure (e.g., collaboration with referral institutions, high-risk population screening, standardised CHD training programs, and chronic disease information systems). (3) Outcome: Evaluated community GPs’ mastery of CHD core knowledge (e.g., disease concepts, diagnostic criteria, treatment principles, management goals, and drug mechanisms). (4) Challenges and Needs: Surveyed GPs’ perceived barriers and priorities in CHD management (details in Supplementary file 2).

This study adopts the accuracy of GPs’ knowledge, rather than patient treatment outcomes, as the outcome variable, based on two primary considerations. First, obtaining reliable CHD treatment outcome data from PHC institutions in Shanghai is highly challenging: (a) electronic health records are fragmented, and standardised cardiovascular disease endpoint indicators are lacking [25, 26]; and (b) high patient mobility leads to considerable loss-to-follow-up bias [27]. Second, GPs’ knowledge directly influences the standardisation of care, serves as a foundational component of PHC quality, and is closely associated with CHD management outcomes. Therefore, it represents a critical indicator for improving healthcare quality.

#### Regional classification criteria and quantitative evaluation indicators

In this study, the classification of PHC institutions into urban and suburban areas was primarily based on Shanghai municipal regulations governing the identification of suburban PHC institutions [28]. Specifically, the suburban scope included all PHC institutions in Chongming, Fengxian, Qingpu, and Jinshan, Songjiang districts, as well as selected areas of Pudong New district (details in Supplementary file 3). According to these regulations, the study included 154 urban PHC institutions and 93 suburban institutions.

To evaluate medical resource allocation and diagnostic capability at the PHC level, two indicators were employed: mention rate and correct response rate. The mention rate is defined as the proportion of institutions that reported being equipped with specific medications or diagnostic tools, calculated as: *Number of institutions equipped with specific medications or devices / Total number of surveyed institutions × 100%*. This indicator reflects the availability and distribution of essential resources, similar to facility readiness metrics used in service capacity assessments [29].The correct response rate, defined as the proportion of institutions correctly identifying the recommended diagnostic or treatment option in knowledge-based scenarios, serves as a proxy for diagnostic capacity and provider knowledge, calculated as: *Number of respondents providing accurate answers / Total number of surveyed personnel× 100%*.This approach aligns with previous health system evaluations that assess institutional knowledge and readiness through simulated case responses or structured questionnaires [30]. Additionally, to determine the ranking of resource indicators, we calculated the overall mention rate for each specific item within its category. This mention rate was defined as the proportion of institutions reporting that they met the predefined standard for that item. Items were then ranked in descending order of their mention rates to highlight the most and least widely available resources across surveyed institutions.

These quantitative indicators objectively reflect the resource allocation status and diagnostic service capabilities of PHC institutions. They provide scientific evidence for interregional comparisons and offer research support for optimising regional health resource allocation.

#### Survey methodology and quality control

From April to May 2024, a questionnaire survey targeting PHC institutions and their GPs was conducted. To ensure scientific validity and accuracy, domain experts were invited to review the questionnaire content prior to formal implementation. A pilot survey was then carried out following the convenience sampling principle, involving two PHC institutions and ten GPs. Ambiguous questions identified during the pilot phase were revised to enhance clarity, resulting in the finalised version of the questionnaire.

The formal questionnaires were distributed electronically via Wenjuanxing (a professional online survey platform), utilising the quality control network of the Shanghai Municipal Clinical Quality Control Centre for General Practice. During pre-testing, we observed that respondents who completed the full questionnaire in under two minutes frequently provided low-quality or patterned responses (e.g., selecting the same option throughout), indicating inattentiveness. This observation aligns with prior studies that have set comparable thresholds to identify insufficient effort responding [31]. Based on these findings, responses with completion times under two minutes or containing missing entries were classified as invalid and excluded from analysis. This rigorous quality control protocol guaranteed the integrity and representativeness of the collected data.

### Statistical analysis

Survey data collected via the Wenjuanxing platform were exported to Excel and subsequently imported into Statistical Package for Social Sciences (SPSS) for Mac (Version 26.0, SPSS, Inc., Chicago, IL, USA) for statistical analysis. During data processing, continuous variables such as years of professional experience were assessed for normality using standard tests. Variables conforming to a normal distribution were described using mean ± standard deviation. Categorical data, including frequencies and proportions of classification variables, were expressed as rates or percentages. Intergroup comparisons were performed using the chi-square (χ²) test to evaluate differences between subgroups, with a *P-value* < 0.05 considered statistically significant. This analytical framework ensured rigorous evaluation of associations and trends within the dataset, aligning with established methodologies for healthcare resource and service capability research.

## Results

### Basic information of PHC institutions

This study conducted a comprehensive survey of PHC institutions across 16 districts in Shanghai, including 247 institutions in total. Among these, 154 institutions (154/247,62.3%) were located in urban areas, while 93 institutions (93/247,37.7%) were situated in suburban regions. A total of 247 institutional questionnaires were distributed, achieving a 100.0% recovery rate, with all returned questionnaires deemed valid, resulting in a 100.0% validity rate.

### Basic information of community GPs

A total of 2,274 questionnaires were distributed to GPs, and 2,112 were returned, yielding a response rate of 92.9%. Of these, 8 questionnaires were completed in fewer than 2 min, and 11 contained missing items, both of which were excluded from the analysis. Consequently, 2,093 valid questionnaires were retained, resulting in an effective response rate of 99.1% (details in Supplementary file 2). Among the 2,093 GPs surveyed, 68.1% (1,426/2,093) were from urban areas, and 31.9% (667/2,093) were from suburban areas. Detailed demographic and professional characteristics are summarised in Table 1.

**Table 1:** Sociodemographic characteristics of 2093 community GPs surveyed in Shanghai, [*n* (%)]

Category	Community GPs (*n* = 2,093)
**Gender**	
Male	630 (30.1)
Female	1,463 (69.9)
**Professional title**	
Intermediate or below	1,561 (74.6)
Above intermediate	532 (25.4)
**Education background**	
Bachelor’s degree or below	1,820 (87.0)
Above bachelor’s degree	273 (13.0)
**Work experience category**	
Short-term (< 15 years)	991 (47.3)
Long-term (≥ 15 years)	1102 (52.7)
**Work area**	
Urban district	1,426 (68.1)
Suburban district	667 (31.9)
**Participation in GP standardized training**	
Yes	1,224 (58.5)
No	869 (41.5)

Note: GP: General practitioner

### Findings on the quality of CHD diagnosis and treatment and management capacity

#### Resource allocation

Human resource allocation: Among institutions offering specialised outpatient services for CHD, the majority adopted general-specialty integrated care teams (74.8%, 119/159). However, across all surveyed institutions, the availability rate of community-based cardiac rehabilitation physiotherapists remained low at 14.6% (36/247). Medications and equipment: Key medications (e.g., aspirin, statins) achieved 100% coverage in all institutions. Core diagnostic equipment (e.g., electrocardiogram machines, defibrillators) demonstrated high availability (> 97.0%). In contrast, cardiac rehabilitation equipment showed limited availability, with only 8.5% (21/247) of institutions equipped with such resources. Detailed comparative data are provided in Table 2.

**Table 2:** Overall allocation of resources for CHD diagnosis and treatment in 247 PHC institutions in Shanghai, [*n* (%)]

Category	Specific item	Overall mention rate (*n* = 247)	Ranking
Outpatient staffing	General-specialty integrated team	119/159 (74.8)	1
	Cardiovascular Specialist from General Hospital	27/159 (17.0)	2
	Local community GPs	13/159 (8.2)	3
Other staffing	Cardiac resuscitation emergency team	244 (98.8)	1
	CHD rehabilitation therapist	36 (14.6)	2
Examination items & equipment	Electrocardiogram (ECG)	246 (99.6)	1
	Myocardial injury biomarkers	230 (93.1)	2
	24-hour dynamic ECG	217 (87.9)	3
	Cardiac echocardiography	142 (57.5)	4
	ECG stress test	11 (4.5)	5
Treatment equipment	Cardiac defibrillator	240 (97.2)	1
	Post-resuscitation treatment equipment	94 (38.1)	2
	CHD rehabilitation equipment	21 (8.5)	3
	Emergency cardiac pacemaker support equipment	20 (8.1)	4
Medication availability	Aspirin	247 (100.0)	1
	Statins	247 (100.0)	1
	Beta-blockers	247 (100.0)	1
	ACEI/ARB	247 (100.0)	1
	Dihydropyridine calcium channel blockers	246 (99.6)	2
	Clopidogrel or ticagrelor	245 (99.2)	3
	Nitrates	239 (96.8)	4
	Trimetazidine	224 (90.7)	5
	Non-dihydropyridine calcium channel blockers	198 (80.2)	6
	Ezetimibe	183 (74.1)	7

Note: CHD: Coronary heart disease; ACEI: Angiotensin-Converting Enzyme Inhibitor; ARB: Angiotensin II Receptor Blocker

#### Initiatives related to the quality of CHD diagnosis and treatment and management

Implementation of disease management programs: PHC institutions demonstrated robust coverage of basic services. 91.5% (226/247) had established referral coordination mechanisms with tertiary hospitals, and 89.0% (186/209) achieved annual screening coverage for high-risk populations. However, patient self-management programs were notably underdeveloped, with only 18.6% (46/247) of institutions operating CHD self-management groups.

Quality control system development: While 81.0% (200/247) of institutions conducted standardised CHD diagnosis and treatment training—indicating gradual improvement in continuing medical education—quality monitoring mechanisms lagged significantly. Only 47.8% (118/247) implemented standardised clinical practice audits, and merely 27.1% adopted dynamic tracking systems for CHD management records.

Informatics capacity building: Foundational information system infrastructure remained underdeveloped. Only 26.7% (66/247) of institutions deployed dedicated CHD chronic disease management systems, with 21.2% (14/66) achieving internal system integration. Regional coordination mechanisms were particularly deficient, as only 13.6% (9/66) enabled data interoperability with regional healthcare platforms. Detailed data are presented in Table 3.

**Table 3:** Overall status of CHD diagnosis and treatment management in 247 PHC institutions in Shanghai, [*n* (%)]

Category	Specific item	Overall mention rate	Ranking
Disease management	Collaborative higher-level referral institution	226 (91.5)	1
	High-risk population screening	186/209 (89.0)	2
	Standardized CHD diagnosis/training programs	200 (81.0)	3
	Follow-up for CHD patients	157/209 (75.1)	4
	Dedicated CHD outpatient clinic	159 (64.4)	5
	CHD Self-management groups	46 (18.6)	6
Quality control	Implementation rate of standardized CHD training	200 (81.0)	1
	Compliance rate of CHD diagnosis guidelines	118 (47.8)	2
	CHD management records/archives	67 (27.1)	3
Informatization	CHD chronic disease information system	66 (26.7)	1
	Interoperability with internal HIS system	14/66 (21.2)	2
	Interoperability with regional HIS systems	9/66 (13.6)	3

Note: HIS: Health information systems

#### Analysis of GPs’ knowledge competency in CHD

**Fig. 1:**
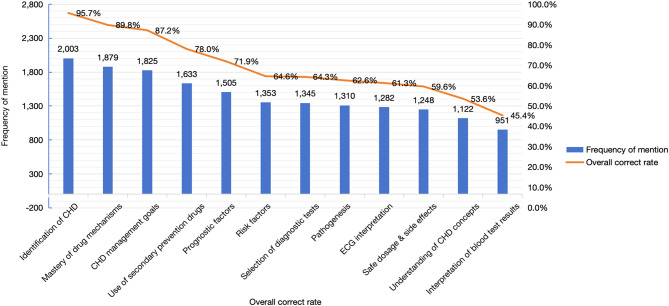
Overall correctness of CHD knowledge responses among 2093 community GPs in Shanghai

GPs demonstrated strong proficiency in CHD differential diagnosis (95.7% accuracy, 2,003/2,093) and knowledge of medication mechanisms (89.8% accuracy, 1,879/2,093). However, significant gaps were observed in conceptual understanding of CHD (53.6% accuracy, 1,122/2,093) and interpretation of blood test results (45.4% accuracy, 951/2,093), indicating areas requiring targeted educational interventions. Specific results are detailed in Fig. 1.

The overall mean accuracy rate for CHD-related knowledge was 66.67% ± 15.90% (*mean ± SD*). No statistically significant difference was observed between urban and suburban practitioners in mean accuracy rates (66.64% ± 16.03% vs. 66.72% ± 15.66%, *t = -0.107*, *p = 0.915*), as summarised in Table 4.

**Table 4:** Mean correctness of CHD knowledge responses by GPs in urban and suburban communities in Shanghai, [*n* (%)] ($\bar X$± s)

Category	Urban mean correct rate (*n* = 1,426)	Suburban mean correct rate (*n* = 667)	Overall mean correct rate (*n* = 2,093)	t-value	*p*-value
Knowledge of CHD	66.64 ± 16.03	66.72 ± 15.66	66.67 ± 15.90	-0.107	0.915

#### Challenges and needs in community-based CHD management

**Fig. 2:**
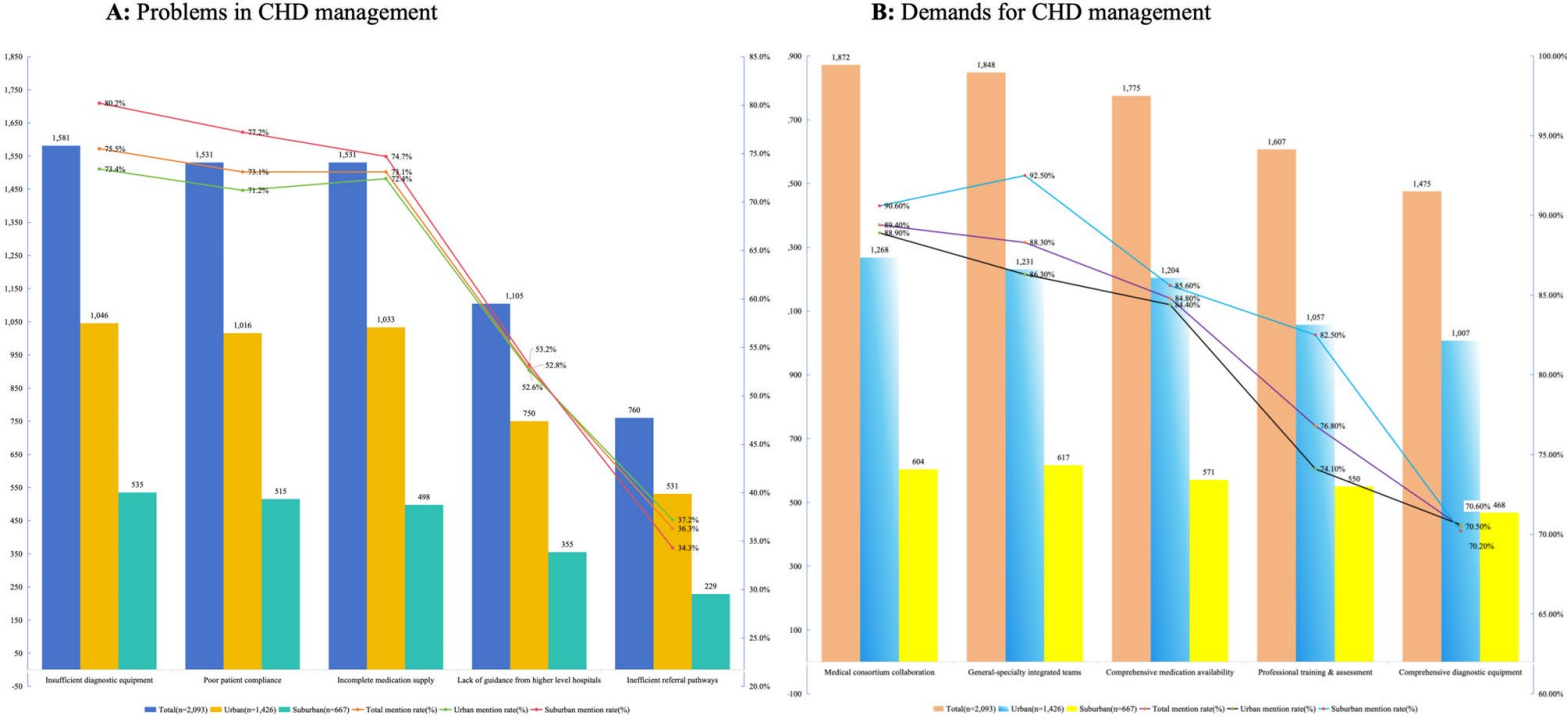
Problems and needs of community GPs in Shanghai for the implementation of CHD management programs

In a Shanghai community-based CHD management program involving GPs, the most frequently reported issue by urban and suburban GPs was the lack of CHD screening equipment (75.5%, 1,581/2,093). Meanwhile, the most critical needs identified by GPs were medical consortium collaboration (89.4%,1,872/2,093) and comprehensive-specialist team development (88.3%, 1,848/2,093), as detailed in Fig. 2.

## Discussion

This study conducted an in-depth analysis of the quality of CHD diagnosis, treatment and management capabilities in PHC institutions in Shanghai. The findings suggest that while these institutions have made notable progress—particularly in medication availability, emergency equipment readiness, and referral mechanisms—critical gaps remain. Urgent improvements are needed in areas such as rehabilitation services, informatisation infrastructure, and GP training. The following sections elaborate on these strengths and weaknesses from the perspectives of diagnostic and treatment quality, as well as management capacity, and propose corresponding improvement strategies.

### Strengths and weaknesses in the quality of community-based CHD diagnosis and treatment

PHC institutions in Shanghai demonstrate distinct advantages in CHD diagnosis and treatment quality, particularly in emergency equipment allocation. The study reveals a 97.2% coverage rate of cardiac defibrillators, ensuring effective emergency care during acute CHD episodes. Additionally, sufficient medication resources guarantee timely treatment during acute phases. Nevertheless, despite adequate emergency equipment and pharmacological interventions, significant deficiencies remain in comprehensive patient management, especially in rehabilitation services. The limited rehabilitation capacity is primarily reflected in the low rehabilitation equipment availability (8.5%). This disparity may stem from policy priorities favoring acute-phase treatment over chronic disease management and rehabilitation, compounded by financial constraints in primary institutions. Substantial evidence confirms that cardiac rehabilitation improves cardiopulmonary health, quality of life, and reduces mortality and hospitalisation rates [32–34]. Consequently, inadequate rehabilitation services not only hinder patient recovery but also increase referrals to tertiary hospitals, exacerbating healthcare resource burdens [35, 36].

To enhance CHD rehabilitation capabilities in Shanghai’s PHC institutions, international best practices offer valuable guidance. For example, the 2024 joint guidelines issued by the American Heart Association (AHA) and the American Association of Cardiovascular and Pulmonary Rehabilitation (AACVPR) identify key components of effective cardiac rehabilitation programs: (1) individualised treatment plans, (2) multidisciplinary care teams, (3) integration of virtual rehabilitation technologies, (4) intensified nutritional and weight management interventions, (5) comprehensive psychosocial support, and (6) optimised exercise regimens [37]. These strategies serve as critical references for improving CHD rehabilitation systems in Shanghai.

### Opportunities and challenges in community-based CHD management capacity

Shanghai’s PHC institutions exhibit mature practices in high-risk population screening and referral mechanisms. Efficient patient transfers to specialised hospitals during critical conditions ensure timely acute-phase treatment [38]. Simultaneously, effective screening enables early identification and intervention for potential CHD cases [12]. However, notable deficiencies persist in patient self-management support and HIS. The study reveals only 18.6% implementation of standardised CHD self-management programs, reflecting systemic weaknesses in patient empowerment and education. This suggests a service model that prioritises screening over intervention—highlighting a critical gap in addressing the long-term needs of chronic disease management and underscoring the underdevelopment of comprehensive self-management frameworks. This weakness correlates with insufficient emphasis on behavioral interventions and health education in primary institutions, where performance evaluations prioritize acute care over long-term health management. Concurrently, inadequate patient education hinders active participation in daily health management, compromising self-management effectiveness. Additionally, only 47.8% (118/247) of institutions conducted standardised clinical practice audits, and merely 27.1% had adopted dynamic tracking systems for managing CHD records. These findings underscore significant deficiencies in closed-loop quality control systems. Furthermore, informatisation limitations further constrain management capacity. International experiences demonstrate advanced primary care models for CHD in developed countries. Australia’s quality improvement interventions optimised care processes, enhanced clinician engagement, and improved preparedness for public health emergencies in chronic disease management [39]. Germany’s integrated disease management programs synergise patient education, personalised treatment, and multidisciplinary collaboration, significantly improving clinical outcomes and quality of life while reducing complications and costs [40]. Italy’s multicentre longitudinal studies systematically analysed self-care capacities and influencing factors using standardised tools, informing precision intervention strategies [41]. These models provide valuable references for optimising service workflows, multidisciplinary collaboration, and self-management assessments in China.

Notably, only 26.7% of Shanghai’s primary institutions utilise chronic disease information management systems for CHD, and interoperability between hospital and regional health information systems (HIS) remains limited—highlighting the persistence of information silos within the healthcare infrastructure. This deficiency likely originates from insufficient funding and technical support, hindering informatisation development. Inefficient data sharing impedes health information integration across institutions, adversely affecting care quality and efficiency. Research confirms that robust HIS can transcend geographical barriers and optimise resource allocation, particularly enhancing care accessibility in underserved areas and improving cardiovascular disease management [42]. Therefore, prioritising self-management capacity-building through regular health education and patient empowerment initiatives is imperative. Concurrently, accelerating informatisation infrastructure development—particularly through increased investments in interoperable regional systems—will substantially enhance service efficiency and quality [43].

### Need for enhancing knowledge and competence of community GPs

The study reveals that while GPs in PHC institutions demonstrate solid foundational knowledge in basic CHD management, deficiencies persist in critical domains such as ECG interpretation, safe medication dosing, and understanding prognostic factors for CHD patients. These gaps highlight shortcomings in knowledge updates and specialised training at the primary care level. Although cardiology-specific training programs exist, limited funding and technical support in primary institutions constrain the scope and frequency of such training, hindering timely updates in key areas [44]. Furthermore, rapid advancements in CHD treatment and management strategies outpace the capacity of primary institutions to adapt, exacerbating knowledge gaps [45]. To address these issues, targeted training programs focusing on updated clinical guidelines and practical skills should be prioritized [46]. Hybrid approaches combining online modules, hands-on workshops, and expert-led seminars could strengthen GPs’ competencies in weak areas. Additionally, optimising assessment mechanisms—through regular knowledge evaluations and performance benchmarking—may incentivise continuous professional development and improve service quality.

Notably, the study found no significant difference in CHD knowledge accuracy between urban and suburban GPs (*p* = 0.915). This may reflect Shanghai’s standardised training system and the integration of medical consortium resources, which promote uniform knowledge updates across regions. However, potential self-selection bias among voluntarily participating GPs (e.g., those with higher competence may be more inclined to engage) might obscure actual disparities. Future studies should incorporate stratified variables (e.g., training frequency, years of primary care experience) to better elucidate regional characteristics.

### Comprehensive recommendations and improvement strategies

While Shanghai’s PHC institutions have achieved notable progress in the diagnosis, treatment, and acute management of CHD, substantial improvements remain necessary in rehabilitation services, informatisation, and GP capabilities. To address these systemic gaps, a multifaceted strategy is proposed: (1) strengthening rehabilitation infrastructure through increased investment in equipment and specialised personnel to support continuous, long-term care [47]; (2) developing structured self-management programs that incorporate intensive health education to enhance patient engagement [48]; (3) prioritising the implementation of interoperable HIS to improve data integration and care coordination [49]; and (4) advancing competency-based GP training through blended learning models and performance-based incentives [50]. Collectively, these strategies aim to enhance the capacity of PHC institutions to provide evidence-based, patient-centred care, thereby promoting comprehensive CHD management and alleviating pressures on the broader healthcare system.

### Limitations

This research has several limitations. First, as a cross-sectional study, its findings reflect the current status of CHD care in Shanghai’s primary institutions, with inherent geographical and temporal constraints. Generalizability to other regions or healthcare tiers requires further validation. Second, although the accuracy of GPs’ knowledge is a key entry point for healthcare quality improvement, its use as a proxy indicator cannot fully reflect actual patient outcomes. The results of the study can only indirectly infer the association between the medical process and potential outcomes, but cannot directly verify the real impact of knowledge level on the quality of patient survival or clinical endpoints. Third, the study relied on self-reported data from institutions and providers, which may be subject to social desirability bias—potentially leading to over-reporting of favorable practices or resource availability. Finally, the absence of patient behavior data and multi-stakeholder collaboration metrics limits a holistic understanding of CHD management challenges. Nevertheless, this community-based analysis provides a valuable reference for future research.

In the future, it is recommended that after a unified and standardised CHD registration system for PHC institutions has been established, a longitudinal study should be conducted in conjunction with data on the long-term outcome of patients, so as to clarify the regression of CHD patients in terms of treatment and management. Furthermore, future research could explore whether adherence varies according to individual GPs’ years of experience and educational background. Such analyses could provide deeper insights into potential provider-level factors influencing guideline compliance and inform targeted training or quality improvement initiatives. And by combining patient satisfaction surveys and cost-effectiveness analyses, a comprehensive assessment of the management and treatment capacity of PHC organisations for CHD can be made, providing a more scientific and rigorous basis for policy formulation and resource allocation.

## Conclusions

Community CHD management in Shanghai is characterised by “strong foundation but weak rehabilitation and informatisation” in terms of resource allocation, with a notable imbalance in the knowledge structure of GPs. It is recommended to improve the rehabilitation service system, build a regional information sharing platform, and strengthen the knowledge and ability of CHD through stratified training, in order to promote the quality of community CHD diagnosis and treatment and the development of high-quality management ability.

## Supplementary Information

Below is the link to the electronic supplementary material.

Supplementary Material 1

Supplementary Material 2

Supplementary Material 3

## Data Availability

The data are available from the corresponding author upon reasonable request.

## Abbreviations

CHD: Coronary heart disease
PHC: Primary health care
GP: General practitioner
SPO: Structure-Process-Outcome
QOF: Quality and Outcomes Framework
ACEI: Angiotensin-Converting Enzyme Inhibitor
ARB: Angiotensin II Receptor Blocker
AHA: American Heart Association
ARIC: Atherosclerosis Risk in Communities
COPD: Chronic obstructive pulmonary disease
SPSS: Statistical Package for Social Sciences
ECG: Electrocardiogram
AACVPR: American Association of Cardiovascular and Pulmonary Rehabilitation
HIS: Health information systems
