# Supplementary material for: Management capacity for stable coronary heart disease in Shanghai community medical institutions: a cross-sectional study
Source: BMC Health Serv Res. 2025 Oct 7;25:1318. doi: 10.1186/s12913-025-13486-y (PMC12506379; doi:10.1186/s12913-025-13486-y)
Supplement: Supplementary file 1 — Supplementary Material 1 [file 12913_2025_13486_MOESM1_ESM.docx]

**Supplementary file 1. Questionnaire on the current status of diagnosis and treatment of**

**CHD in community health service centres in Shanghai**

**I. Basic institutional information**

1. The district in which your organization is located is: [Single choice]

| Options | Subtotal | Proportions |
| --- | --- | --- |
| Jing'an district | 15 | 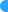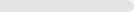6.07% |
| Jiading district | 13 | 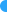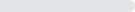5.26% |
| JinShan District | 11 | 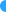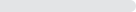4.45% |
| Yangpu district | 12 | 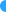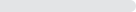4.86% |
| Xuhui district | 12 | 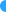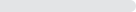4.86% |
| Fengxian district | 17 | 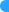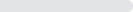6.88% |
| Huangpu District | 10 | 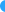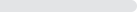4.05% |
| Hongkou district | 8 | 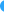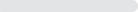3.24% |
| Baoshang district | 19 | 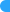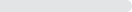7.69% |
| Changning district | 10 | 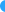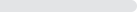4.05% |
| Songjiang district | 18 | 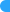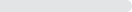7.29% |
| Minhang district | 14 | 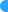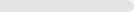5.67% |
| Chongming District | 18 | 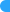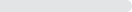7.29% |
| Qingpu district | 12 | 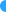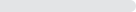4.86% |
| Putuo district | 12 | 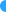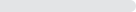4.86% |
| Pudong district | 46 | 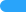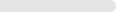18.62% |
| Number of valid entries for this question | 247 |  |

2. The name of your organization is: ________ community health service center [fill in the blank]

**II. Current status of coronary heart disease (CHD) diagnosis and treatment**

1. Does your organization routinely diagnose and manage CHD? [Single choice]

| options | Subtotal | Proportions |
| --- | --- | --- |
| A. Yes | 209 | 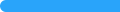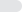84.62% |
| B. None | 38 | 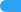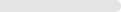15.38% |
| Number of valid entries for this question | 247 |  |

2. What is the specific management program for CHD at your organization? [Multiple Choice]

| Options | Subtotal | Proportions |
| --- | --- | --- |
| A. Screening of high-risk groups (including medical check-ups for the elderly) | 186 | 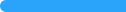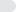89% |
| B. Recognition and diagnosis | 199 | 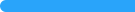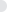95.22% |
| C. Two-way referral | 200 | 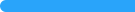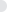95.69% |
| D. Follow-up | 157 | 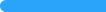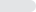75.12% |
| E. Other support work (self-management, community management aspects) | 65 | 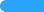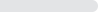31.1% |
| Number of valid entries for this question | 209 |  |

3. Does your organization have a clinic specializing in "CHD" or a "cardiology all-specialty" clinic? [Single choice]

| Options | Subtotal | Proportions |
| --- | --- | --- |
| A. Yes | 159 | 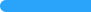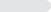64.37% |
| B. No | 88 | 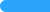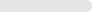35.63% |
| Number of valid entries for this question | 247 |  |

4. What are the main personnel of your medical institution who carry out specialized clinics for CHD or comprehensive clinics for cardiology? [Single choice]

| Options | Subtotal | Proportions |
| --- | --- | --- |
| A. General practitioners in the community | 12 | 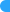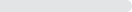7.55% |
| B. Cardiologists in general hospitals | 27 | 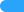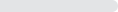16.98% |
| C. General practitioners in the community in conjunction with specialists from general hospitals (all-specialty combined team) | 119 | 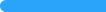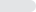74.84% |
| D. Other | 1 | 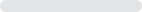0.63% |
| Number of valid entries for this question | 159 |  |

5. How often does your organization conduct a combined coronary or cardiology all-specialty clinic? [Single choice]

| Options | Subtotal | Proportions |
| --- | --- | --- |
| A. Conducted as a regular daily clinic visit | 6 | 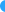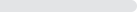3.77% |
| B. Weekly | 91 | 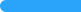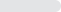57.23% |
| C. On average every two weeks | 51 | 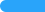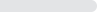32.08% |
| D. On average once a month | 11 | 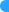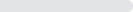6.92% |
| E. Less than once a month | 0 | 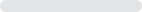0% |
| Number of valid entries for this question | 159 |  |

6. What are the main ancillary tests performed in your organization for coronary artery disease and its complications? [Multiple Choice]

| Options | Subtotal | Proportions |
| --- | --- | --- |
| A. Electrocardiograms (ECG) | 246 | 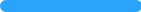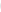99.6% |
| B. Myocardial injury markers: cardiac troponin I or T, creatine kinase (CK) and isozymes (CK-MB) | 230 | 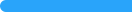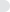93.12% |
| C. Echocardiography of the heart | 142 | 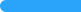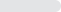57.49% |
| D. Multilayer spiral CT coronary artery imaging (CTA) | 9 | 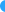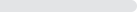3.64% |
| E. Electrocardiogram loading test | 11 | 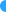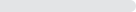4.45% |
| F. ECG Continuous Dynamic Detection (Holter) | 217 | 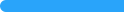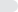87.85% |
| G. Radionuclide examinations (nuclear myocardial imaging and loading test, radionuclide cardiac chambers imaging, positron emission tomography myocardial imaging PET) | 0 | 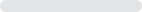0% |
| H. Coronary angiography (CAG) | 1 | 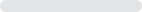0.4% |
| I. Other | 0 | 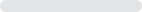0% |
| Number of valid entries for this question | 247 |  |

7. What are the main medications equipped (including extended prescriptions) for the treatment of CHD and its complications in your organization? [Multiple choice]

| Options | Subtotal | Proportions |
| --- | --- | --- |
| A. Aspirin or indobufen | 247 | 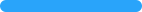100% |
| B. Clopidogrel hydrogencarbamate or tegretol sulfate | 245 | 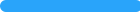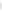99.19% |
| C. Beta receptor antagonists (metoprolol, bisoprolol) | 247 | 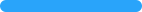100% |
| D. ACEI or ARB (captopril, enalapril, perindopril, benadryl, etc.) | 247 | 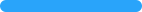100% |
| E. Calcium channel blockers (non-dihydropyridines): verapamil, diltiazem | 198 | 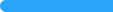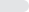80.16% |
| F. Calcium channel blockers (dihydropyridines): nifedipine, amlodipine | 246 | 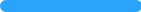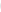99.6% |
| G. Trimetazidine | 224 | 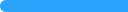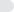90.69% |
| H. Ezetimibe | 183 | 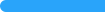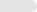74.09% |
| I. Nitrate esters (isosorbide mononitrate, isosorbide dinitrate, nitroglycerin) | 239 | 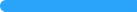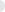96.76% |
| J. Statins (simvastatin, atorvastatin, pravastatin, etc.) | 247 | 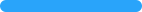100% |
| K. Nitrate esters (isosorbide mononitrate, isosorbide dinitrate, nitroglycerin) | 239 | 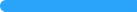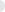96.76% |
| L. Other drugs, please specify | 4 | 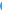1.62% |
| Number of valid entries for this question | 247 |  |

8. What measures and equipment are in place in your organization to resuscitate patients with sudden cardiac arrest? (EMS team and equipment) [Multiple choice]

| Options | Subtotal | Proportions |
| --- | --- | --- |
| Availability of cardiopulmonary resuscitation (CPR) equipment and staff with relevant training | 244 | 98.79% |
| Possession of electrocardiogram (ECG) equipment and related diagnostic software | 244 | 98.79% |
| Has a cardiac defibrillator (AED) | 240 | 97.17% |
| Provision of emergency cardiac pacing support equipment | 20 | 8.1% |
| Transportation is available for rapid transfer of patients to hospitals | 66 | 26.72% |
| Emergency Hotline and Emergency Medical Assistance Service available. | 55 | 22.27% |
| Post-resuscitation therapy equipment and medications for cardiac arrest patients available | 94 | 38.06% |
| Conducting relevant first aid drills and training on a regular basis to improve the first aid level of the medical team | 218 | 88.26% |
| Number of valid entries for this question | 247 |  |

9. Does your organization have a record or file for the management of patients with coronary artery disease (including sound coronary artery disease-related content is sufficient)? [Single choice]

| Options | Subtotal | Proportions |
| --- | --- | --- |
| A. Yes | 67 | 27.13% |
| B. No | 180 | 72.87% |
| Number of valid entries for this question | 247 |  |

10. How long does it typically take to update your organization's records or files for the management of patients with coronary artery disease? [Single choice]

| Options | Subtotal | Proportions |
| --- | --- | --- |
| A. 1 time per week | 2 | 2.99% |
| B. 1 per month | 5 | 7.46% |
| C. 1 per quarter | 12 | 17.91% |
| D. Updated at the time of the patient's visit | 42 | 62.69% |
| E. Uncertainty about the timing of the update | 6 | 8.96% |
| Number of valid entries for this question | 67 |  |

11.Does your organization have any supervision over prescription practices and treatment standardization for patients with coronary artery disease? [Single choice]

| Options | Subtotal | Proportions |
| --- | --- | --- |
| A. Yes | 118 | 47.77% |
| B. No | 129 | 52.23% |
| Number of valid entries for this question | 247 |  |

12. How often does your organization monitor the prescribing and standardization of care for patients with coronary artery disease? [Single choice]

| Options | Subtotal | Proportions |
| --- | --- | --- |
| A. 1 time per week | 3 | 2.54% |
| B. 1 per month | 59 | 50% |
| C. 1 per quarter | 25 | 21.19% |
| D. Semi-annually | 8 | 6.78% |
| E. Uncertainty about the timing of inspections | 23 | 19.49% |
| Number of valid entries for this question | 118 |  |

13. Does your organization provide training on standardized diagnosis and management of CHD for general practitioners? [Single choice]

| Options | Subtotal | Proportions |
| --- | --- | --- |
| A. Yes | 200 | 80.97% |
| B. No | 47 | 19.03% |
| Number of valid entries for this question | 247 |  |

14. How is the frequency of training related to the standardized diagnosis and management of coronary artery disease among general practitioners in your organization, and what is the typical duration of such training? [Single choice]

| Options | Subtotal | Proportions |
| --- | --- | --- |
| A. 1 time per week | 1 | 0.5% |
| B. 1 per month | 2 | 1% |
| C. 1 per quarter | 21 | 10.5% |
| D. Semi-annually | 36 | 18% |
| E. Uncertainty about the timing of training | 140 | 70% |
| Number of valid entries for this question | 200 |  |

15. Does your organization have a collaborative upper-level referral facility for coronary artery disease? [Single choice]

| Options | Subtotal | Proportions |
| --- | --- | --- |
| A. Yes | 226 | 91.5% |
| B. No | 21 | 8.5% |
| Number of valid entries for this question | 247 |  |

16. Does your organization have the equipment and technology for follow-up rehabilitation of patients with coronary artery disease? [Single choice]

| Options | Subtotal | Proportions |
| --- | --- | --- |
| A. Availability of special rehabilitation equipment for patients with CHD | 21 | 8.5% |
| B. Availability of specialized rehabilitation physiotherapists | 36 | 14.57% |
| C. Availability of specialized rehabilitation techniques for patients with CHD | 2 | 0.81% |
| D. Insufficient experience in rehabilitation services for patients with CHD | 133 | 53.85% |
| E. Follow-up rehabilitation services for patients with CHD have not yet been considered | 54 | 21.86% |
| F. Other, please specify | 1 | 0.4% |
| Number of valid entries for this question | 247 |  |

17. What challenges does your organization face in managing CHD? [Multiple Choice]

| Options | Subtotal | Proportions |
| --- | --- | --- |
| A. Inadequate testing and inspection equipment | 178 | 72.06% |
| B. Inadequate provision of therapeutic drugs | 107 | 43.32% |
| C. Inadequate physician capacity | 173 | 70.04% |
| D. Lack of two-way referral pathways to higher level hospitals | 52 | 21.05% |
| E. Lack of experts to mentor downstream communities | 95 | 38.46% |
| F. Other, please fill in | 5 | 2.02% |
| Number of valid entries for this question | 247 |  |

18. Problems and suggestions: the demand for community health centers to carry out CHD dynamic diagnosis and treatment and improvement suggestions? [fill in the blank]

19. Does your organization conduct and support a community self-management group for CHD? [Single choice]

| Options | Subtotal | Proportions |
| --- | --- | --- |
| A. Yes | 46 | 18.62% |
| B. No | 201 | 81.38% |
| Number of valid entries for this question | 247 |  |

20. Does your organization have an information management system for CHD? [Single choice]

| Options | Subtotal | Proportions |
| --- | --- | --- |
| A. No | 181 | 73.28% |
| B. Yes | 66 | 26.72% |
| Number of valid entries for this question | 247 |  |

21. Is your organization's coronary information management system interconnected with your hospital's HIS system? [Single choice]

| Options | Subtotal | Proportions |
| --- | --- | --- |
| A. Yes | 14 | 21.21% |
| B. No | 52 | 78.79% |
| Number of valid entries for this question | 66 |  |

22. Is your organization's CHD information management system interconnected with the HIS system of the healthcare organizations in the regional medical association? [Single choice]

| Options | Subtotal | Proportions |
| --- | --- | --- |
| A. Yes | 9 | 13.64% |
| B. No | 57 | 86.36% |
| Number of valid entries for this question | 66 |  |
