# Supplementary material for: Management capacity for stable coronary heart disease in Shanghai community medical institutions: a cross-sectional study
Source: BMC Health Serv Res. 2025 Oct 7;25:1318. doi: 10.1186/s12913-025-13486-y (PMC12506379; doi:10.1186/s12913-025-13486-y)
Supplement: Supplementary file 2 — Supplementary Material 2 [file 12913_2025_13486_MOESM2_ESM.docx]

**Supplementary file 2. Questionnaire on the status of general practitioners in Shanghai**

**I. Basic personal information for general practitioners (GPs)**

1. The district in which your organization is located is: [Single choice]

| Options | Subtotal | Proportions |
| --- | --- | --- |
| Jing'an district | 95 | 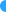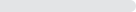4.54% |
| Jiading district | 158 | 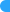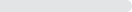7.55% |
| Jinshan district | 79 | 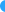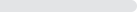3.77% |
| Yangpu district | 96 | 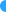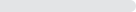4.59% |
| Xuhui district | 103 | 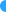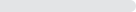4.92% |
| Fengxian district | 114 | 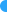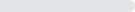5.45% |
| Huangpu district | 55 | 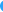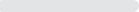2.63% |
| Hongkou district | 52 | 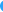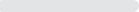2.48% |
| Baoshang district | 125 | 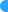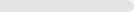5.97% |
| Changning district | 43 | 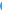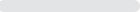2.05% |
| Songjiang district | 161 | 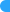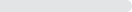7.69% |
| Minhang district | 216 | 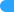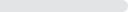10.32% |
| Chongming district | 78 | 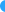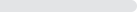3.73% |
| Qingpu district | 106 | 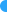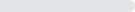5.06% |
| Putuo district | 93 | 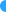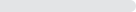4.44% |
| Pudong district | 519 | 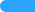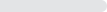24.8% |
| Number of valid entries for this question | 2093 |  |

2. The name of your organization is: ________ Community Health Service Center [fill in the blank]

Fill-in-the-blank data can be obtained by downloading the detailed data.

3. What is your gender? [Single choice]

| Options | Subtotal | Proportions |
| --- | --- | --- |
| A. Male | 630 | 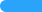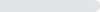30.1% |
| B. Female | 1463 | 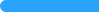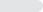69.9% |
| Number of valid entries for this question | 2093 |  |

4. How many years have you been practicing general practice? ____ [fill in the blanks]

5. What is your highest medical-related title? [Single choice]

| Options | Subtotal | Proportions |
| --- | --- | --- |
| A. Primary | 247 | 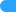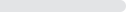11.8% |
| B. Intermediate | 1314 | 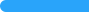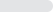62.78% |
| C. Associate and above | 532 | 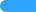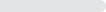25.42% |
| Number of valid entries for this question | 2093 |  |

6. What is your highest medical-related degree? [Single choice]

| Options | Subtotal | Proportions |
| --- | --- | --- |
| A. Specialized | 31 | 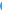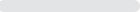1.48% |
| B. Undergraduate | 1789 | 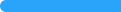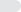85.48% |
| C. Master's Degree and Above | 273 | 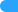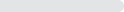13.04% |
| Number of valid entries for this question | 2093 |  |

7. Have you obtained a certificate of residency training in general practice? [Single choice]

| Options | Subtotal | Proportions |
| --- | --- | --- |
| A. Yes | 1224 | 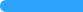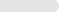58.48% |
| B. No | 869 | 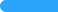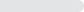41.52% |
| Number of valid entries for this question | 2093 |  |

**II. Current status of coronary heart disease (CHD) diagnosis and treatment**

1. What are the main components of your involvement in the management of patients with coronary artery disease? [Multiple choice]

| Options | Subtotal | Proportions |
| --- | --- | --- |
| A. Diagnosis and screening | 1741 | 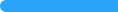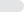83.18% |
| B. Risk assessment and stratified management (follow-up) | 1713 | 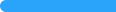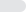81.84% |
| C. Medication | 1946 | 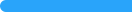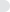92.98% |
| D. Two-way referral | 1780 | 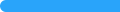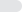85.05% |
| E. Cardiac rehabilitation and lifestyle interventions | 1407 | 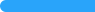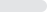67.22% |
| F. Patient education and self-management guidance | 1841 | 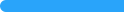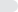87.96% |
| G. Other | 9 | 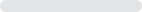0.43% |
| Number of valid entries for this question | 2093 |  |

2. The patient is a middle-aged male who complains of retrosternal tightness and pain every time he walks up the stairs for three months, which is relieved by rest. There are no abnormalities on examination and the electrocardiogram is normal. What is the preferred test to further clarify the diagnosis? [Single choice]

| Options | Subtotal | Proportions |
| --- | --- | --- |
| A. Exercise load test | 1345 | 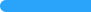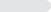64.26% |
| B. Chest X-ray | 12 | 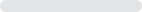0.57% |
| C. Myocardial nuclear imaging | 5 | 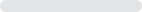0.24% |
| D. Coronary angiography | 535 | 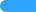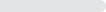25.56% |
| E. Ambulatory electrocardiogram | 196 | 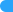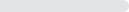9.36% |
| Number of valid entries for this question | 2093 |  |

3. The main difference between acute myocardial infarction and angina pain? [Single choice]

| Options | Subtotal | Proportions |
| --- | --- | --- |
| A. Pain level and duration | 2003 | 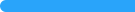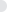95.7% |
| B. The location of the pain is different | 6 | 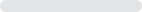0.29% |
| C. The nature of the pain is different | 62 | 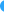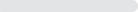2.96% |
| D. Differences in the frequency of pain episodes | 8 | 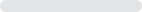0.38% |
| E. Different triggers | 14 | 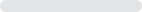0.67% |
| Number of valid entries for this question | 2093 |  |

4. The drug that rapidly terminates an angina attack is? [Single choice]

| Options | Subtotal | Proportions |
| --- | --- | --- |
| A. Nifedipine | 84 | 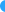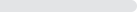4.01% |
| B. Cediran | 19 | 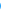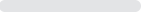0.91% |
| C. Isosorbide nitrate | 1879 | 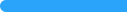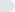89.78% |
| D. Propranolol | 22 | 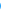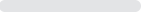1.05% |
| E. Musk Heart Pills | 89 | 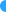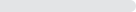4.25% |
| Number of valid entries for this question | 2093 |  |

5. A middle-aged male has been experiencing poststernal compressive pain every day during naps or at night at 2:00 p.m. for the past month, lasting 20 minutes each time and relieved by five minutes of nitroglycerin, with a clinical diagnosis of variant angina pectoris, and the ECG changes during the onset of chest pain should be? [Single choice]

| Options | Subtotal | Proportions |
| --- | --- | --- |
| A. ST-segment elevation in relevant leads | 1282 | 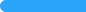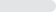61.25% |
| B. ST-segment downshift in the lead in question | 575 | 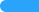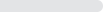27.47% |
| C. No change in the electrocardiogram | 57 | 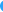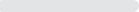2.72% |
| D. T-wave inversion in the relevant leads | 83 | 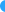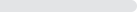3.97% |
| E. Abnormal Q waves in relevant leads | 96 | 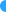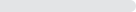4.59% |
| Number of valid entries for this question | 2093 |  |

6. The most important risk factor for atherosclerosis is? [Single choice]

| Options | Subtotal | Proportions |
| --- | --- | --- |
| A. Hypertension | 528 | 25.23% |
| B. Smoking | 168 | 8.03% |
| C. Dyslipidemia | 1353 | 64.64% |
| D. Abnormal blood sugar | 26 | 1.24% |
| E. Obesity | 18 | 0.86% |
| Number of valid entries for this question | 2093 |  |

7. Types of coronary artery disease include? [Single choice]

| Options | Subtotal | Proportions |
| --- | --- | --- |
| A. Angina pectoris | 2016 | 96.32% |
| B. Myocardial infarction | 2012 | 96.13% |
| C. Sudden death | 1327 | 63.4% |
| D. Asymptomatic myocardial ischemia | 1879 | 89.78% |
| E. Ischemic cardiomyopathy | 1833 | 87.58% |
| Number of valid entries for this question | 2093 |  |

8. According to the latest guideline criteria, Low Density Lipoprotein (LDL) in patients with coronary artery disease should be controlled at? [Single choice]

| Options | Subtotal | Proportions |
| --- | --- | --- |
| A.< 2.7 mmol/L | 51 | 2.44% |
| B.< 2.3 mmol/L | 73 | 3.49% |
| C.< 1.8 mmol/L | 1295 | 61.87% |
| D.< 1.6 mmol/L | 144 | 6.88% |
| E.< 1.4 mmol/L | 530 | 25.32% |
| Number of valid entries for this question | 2093 |  |

9. What kind of drug can be given to patients with CHD regardless of their blood lipid levels? [Single choice]

| Options | Subtotal | Proportions |
| --- | --- | --- |
| A. Antiplatelet drugs | 382 | 18.25% |
| B. β-blockers | 15 | 0.72% |
| C. ACEI-type drugs | 26 | 1.24% |
| D. Statins | 1633 | 78.02% |
| E. Nitrates | 37 | 1.77% |
| Number of valid entries for this question | 2093 |  |

10. Which of the following drugs does not improve the prognosis of coronary artery disease? [Single choice]

| Options | Subtotal | Proportions |
| --- | --- | --- |
| A. ACEIs | 194 | 9.27% |
| B. Antiplatelet drugs | 137 | 6.55% |
| C. Statins | 36 | 1.72% |
| D. Nitrates | 1505 | 71.91% |
| E. β-Blockers | 221 | 10.56% |
| Number of valid entries for this question | 2093 |  |

11. What is the drug that is most likely to exacerbate variant angina pectoris? [Single choice]

| Options | Subtotal | Proportions |
| --- | --- | --- |
| A. β-Blockers | 1310 | 62.59% |
| B. Calcium channel blocking sentence | 548 | 26.18% |
| C. Nitrates | 195 | 9.32% |
| D. Antiplatelet drugs | 21 | 1% |
| E. Lipid regulating drugs | 19 | 0.91% |
| Number of valid entries for this question | 2093 |  |

12.Nitroglycerin tablets (0.5mg) are the first-choice drug for the onset of angina pectoris. What is the maximum dosage that can be used within 15 minutes? [Single choice].

| Options | Subtotal | Proportions |
| --- | --- | --- |
| A.1 tablet | 98 | 4.68% |
| B. 2 tablets | 420 | 20.07% |
| C. 3 tablets | 1248 | 59.63% |
| D. 4 tablets | 220 | 10.51% |
| 1. 5 tablets | 107 | 5.11% |
| Number of valid entries for this question | 2093 |  |

13. A patient has a normal ECG presentation but is suspected of having a possible acute or atypical infarction. Which of the following descriptions is correct regarding the timing of the appearance of cardiac enzyme abnormalities during the course of the illness? [Single choice].

| Options (as in computer software settings) | Subtotal | Proportions |
| --- | --- | --- |
| A. Abnormal elevation of creatine kinase (CK) can be detected within 1 hour of onset of disease | 144 | 6.88% |
| B. Creatine kinase isoenzyme (CK-MB) usually peaks within 24 hours after onset of disease | 568 | 27.14% |
| C. Cardiac troponin I (cTnI) begins to rise 3 to 4 hours after the onset of the disease | 951 | 45.44% |
| D. Lactate dehydrogenase (LDH) is abnormally elevated immediately after the onset of the disease | 54 | 2.58% |
| E. Myoglobin (MYO) is elevated within 2 hours of onset of disease | 376 | 17.96% |
| Number of valid entries for this question | 2093 |  |

14. What is your usual referral pathway for patients with coronary artery disease? [Single choice]

| Options | Subtotal | Proportions |
| --- | --- | --- |
| A. Instruct the patient to go to a higher level hospital on their own | 317 | 15.15% |
| B. Instruct the patient to attend the center's all-specialty clinic, and the all-specialty team will be responsible for the upward referral of the patient | 458 | 21.88% |
| C. There is a corresponding contact communication group for rapid peer-to-peer patient referrals. | 829 | 39.61% |
| D. Upward referrals through referral platforms | 464 | 22.17% |
| E. Other | 25 | 1.19% |
| Number of valid entries for this question | 2093 |  |

15. For patients with CHD who have been treated or referred by you, can they be transferred back for continuous follow-up and management by you after surgery or when their condition stabilizes? [Single choice]

| Options | Subtotal | Proportions |
| --- | --- | --- |
| A. Most patients are able to be downgraded after stabilization of the disease and managed with ongoing follow-up by me | 1030 | 49.21% |
| B. Only some of the patients were referred back to the community, and the continuity of follow-up management was more difficult to achieve | 844 | 40.32% |
| C. Few or almost no patients are referred back to the community for ongoing follow-up and management | 203 | 9.7% |
| D. Other situations | 16 | 0.76% |
| Number of valid entries for this question | 2093 |  |

16.What resources or supports do you think would make you better at community management of patients with CHD? [Multiple choice]

| Options | Subtotal | Proportions |
| --- | --- | --- |
| A. Relevant operational training and assessment | 1607 | 76.78% |
| B. Specialized CHD Management Team (All-Specialized Combination) | 1848 | 88.29% |
| C. Comprehensive Therapeutic Drugs | 1775 | 84.81% |
| D. Comprehensive auxiliary inspection equipment | 1475 | 70.47% |
| E. Effective health-care linkages | 1872 | 89.44% |
| F. Assisted diagnosis and treatment decision support or patient risk alerts | 1468 | 70.14% |
| G. Other | 6 | 0.29% |
| Number of valid entries for this question | 2093 |  |

17. What difficulties do you have in the community management of patients with coronary artery disease? [Multiple choice]

| Options | Subtotal | Proportions |
| --- | --- | --- |
| A. Patients have poor adherence to following treatment regimens | 1531 | 73.15% |
| B. Inadequate testing and inspection equipment | 1581 | 75.54% |
| C. Inadequate provision of therapeutic drugs | 1531 | 73.15% |
| D. Insufficient knowledge of their own relevant business | 1136 | 54.28% |
| E. Lack of competence in cardiac rehabilitation | 1291 | 61.68% |
| F. Lack of channels for guidance and communication from higher hospitals (or untimely feedback from channels) | 1105 | 52.8% |
| G. Lack of two-way referral pathways (or inadequate pathways) to higher-level hospitals | 760 | 36.31% |
| H. Other, please fill in | 11 | 0.53% |
| Number of valid entries for this question | 2093 |  |

18. Have you provided guidance and support for self-management in patients with coronary artery disease? [Multiple choice]

| Options | Subtotal | Proportions |
| --- | --- | --- |
| A. No guidance and support has been provided | 260 | 12.42% |
| B. Provide health education and guidance | 1963 | 93.79% |
| C. Participate in the development of individualized treatment and management plans | 1066 | 50.93% |
| D. Assistance in adjusting medications and treatment programs | 1699 | 81.18% |
| E. Providing psychological support and guidance | 1550 | 74.06% |
| F. Coordination of specialists and resources | 997 | 47.63% |
| G. Other, please specify | 1 | 0.05% |
| Number of valid entries for this question | 2093 |  |

19. Have you ever prescribed cardiac rehabilitation for a patient with coronary artery disease? [Multiple choice]

| Options | Subtotal | Proportions |
| --- | --- | --- |
| A. Yes | 307 | 14.67% |
| B. No | 1786 | 85.33% |
| Number of valid entries for this question | 2093 |  |

20.If you have prescribed cardiac rehabilitation for patients with coronary artery disease, indicate what was your primary source for obtaining the relevant evidence-based rationale? [Multiple choice]

| Options | Subtotal | Proportions |
| --- | --- | --- |
| A. Individual self-study and accumulation of experience | 168 | 54.72% |
| B. Guidance and advice from a rehabilitation physician at the same institution | 185 | 60.26% |
| C. Guidance and advice from cardiologists in higher hospitals | 246 | 80.13% |
| D. Shared decision-making by the GP team | 147 | 47.88% |
| E. Shared decision-making by a fully integrated team of specialists | 133 | 43.32% |
| F. Specialized cardiac rehabilitation training courses or seminars | 133 | 43.32% |
| G. Other sources | 2 | 0.65% |
| Number of valid entries for this question | 307 |  |

21. What influences you to prescribe rehabilitation? [Multiple choice]

| Options | Subtotal | Proportions |
| --- | --- | --- |
| A. Lack of appropriate rehabilitation equipment | 1263 | 70.72% |
| B. Lack of knowledge about cardiac rehabilitation | 1265 | 70.83% |
| C. The patient's condition is not suitable for rehabilitation therapy | 392 | 21.95% |
| D. The patient is not interested in or refuses rehabilitation therapy | 730 | 40.87% |
| E. Cardiac rehabilitation services are not available at the healthcare facility where they are located | 942 | 52.74% |
| F. Concerns about the safety and effectiveness of rehabilitation treatment | 467 | 26.15% |
| G. Other reasons. | 14 | 0.78% |
| Number of valid entries for this question | 1786 |  |

22.Questions and Suggestions: What other suggestions do you have for the management of coronary heart disease in the community? [fill in the blank]

Note: Table S1 presents the correspondence between the question numbers from the previous section and the dimensions of GPs' knowledge of CHD as shown in Table 4.

**Table S1 Correspondence between question numbers and dimensions of GPs' Knowledge of CHD**

| **Specific knowledge points** | **The question corresponding to the one mentioned above** |
| --- | --- |
| Identification of CHD | 3 |
| Mastery of drug mechanisms | 4 |
| CHD management goals | 8 |
| Use of secondary prevention drugs | 9 |
| Prognostic factors | 10 |
| Risk factors | 6 |
| Selection of diagnostic tests | 2 |
| Pathogenesis | 11 |
| ECG interpretation | 5 |
| Safe dosage & side effects | 12 |
| Understanding of CHD concepts | 7 |
| Interpretation of blood test results | 13 |
