# Supplementary material for: Management capacity for stable coronary heart disease in Shanghai community medical institutions: a cross-sectional study
Source: BMC Health Serv Res. 2025 Oct 7;25:1318. doi: 10.1186/s12913-025-13486-y (PMC12506379; doi:10.1186/s12913-025-13486-y)
Supplement: Supplementary file 3 — Supplementary Material 3 [file 12913_2025_13486_MOESM3_ESM.docx]

**Supplementary file 3. Classification criteria for urban and suburban**

**community health service centres in Shanghai**

In this study, the urban-suburban classification of Community Health Service Centers (CHSCs) was primarily based on the provisions outlined in the Shanghai Municipal High-Level Evaluation Application Notice for Community Health Services (Hu Wei Ping Shen [2018] No. 2)[1]. Specific classifications were defined as follows:

1. Suburban areas encompass all CHSCs in Chongming District, Fengxian District, Qingpu District, and Jinshan District.
2. The following towns in Songjiang District are classified as suburban areas: Dongjing Town, Jiuting Town, Yexie Town, Shihudang Town, Chedun Town, Xinqiao Town, Xiaokunshan Town, Sigang Town, Xinbang Town, Sheshan Town, and Sijing Town.
3. The following towns in Pudong New Area are classified as suburban areas: Luchaogang, Lingqiao, Huanglou, Liuzao, Jiangzhen, Airport, Caolu, Xuanqiao, Heqing, Shuyuan, Wanxiang, Laogang, Datuan, Nicheng, Sunqiao, Tangzhen, Wanggang, Zhuqiao, Gaodong, Hangtou, Huinan, Xinchang, Chuansha, Hantou, and Hesha.

All CHSCs not explicitly listed as suburban were categorized as urban areas.

**Reference:**

[1] Shanghai Municipal Health Commission, Shanghai Municipal Commission of Development and Reform, et al. (2020-8-21). Notice on the issuance of the "Guidelines for the functions and construction of community health service institutions in Shanghai" [EB/OL]. https://wsjkw.sh.gov.cn/zcjd/20200821/2eb234146ed84ad5b804e7f6a97ee402.html
